# Supplementary material for: Characteristics of Internal Ammonium Loading from Long-Term Polluted Sediments by Rural Domestic Wastewater
Source: Int J Environ Res Public Health. 2019 Nov 22;16(23):4657. doi: 10.3390/ijerph16234657 (PMC6926494; doi:10.3390/ijerph16234657)
Supplement: Supplementary file 1 [file ijerph-16-04657-s001.pdf]

Supplementary Material

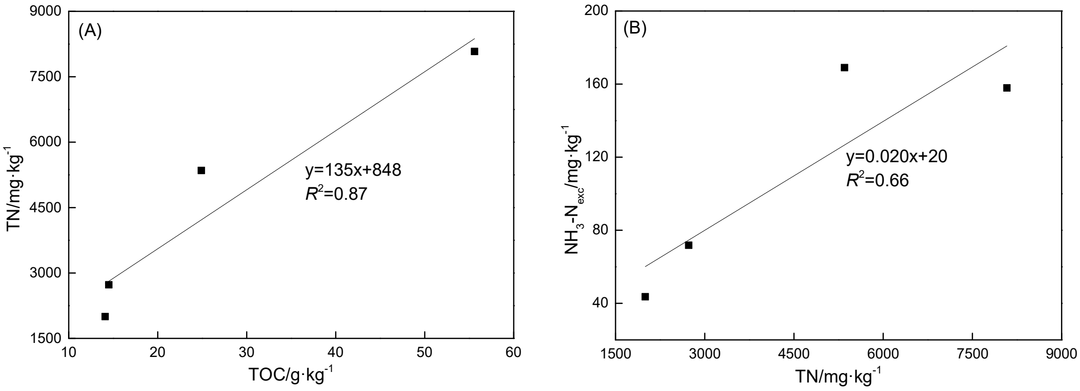

Figure S1. Relationship between TOC (A) and NH_3-N_{exc} (B) of the four selected sediments and TN in the sediments. TN is the total nitrogen content. TOC is the total organic carbon content. NH_3-N_{exc} is the exchangeable ammonium nitrogen content.

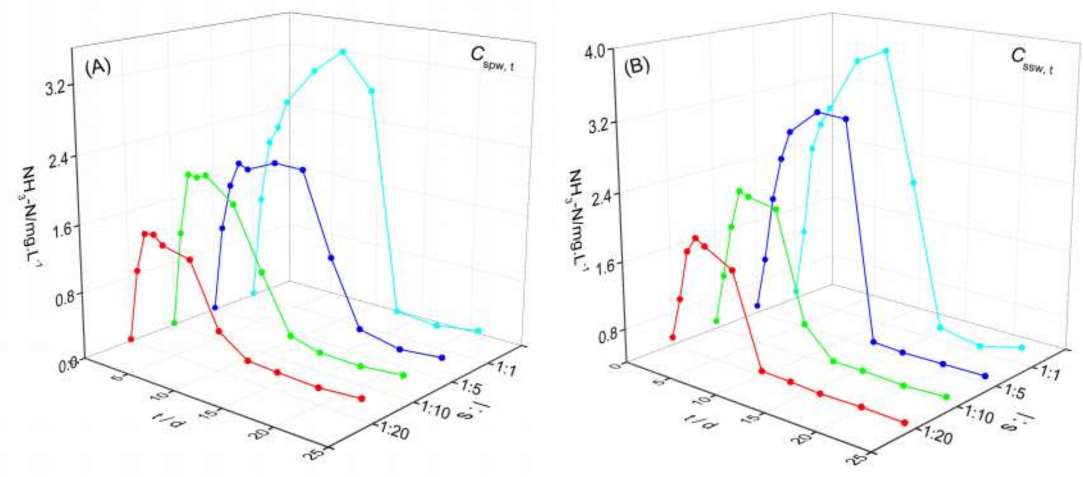

Figure S2. The kinetics of ammonium with sediment (S1) incubation at different sediment-to-water ratios in ultrapure water ($C_{spw,\,t}$) and sample water ($C_{ssw,\,t}$). The controls were the kinetics of ammonium incubation in ultrapure water ($C_{pw,\,t}$) and sample water ($C_{sw,\,t}$) without sediments.

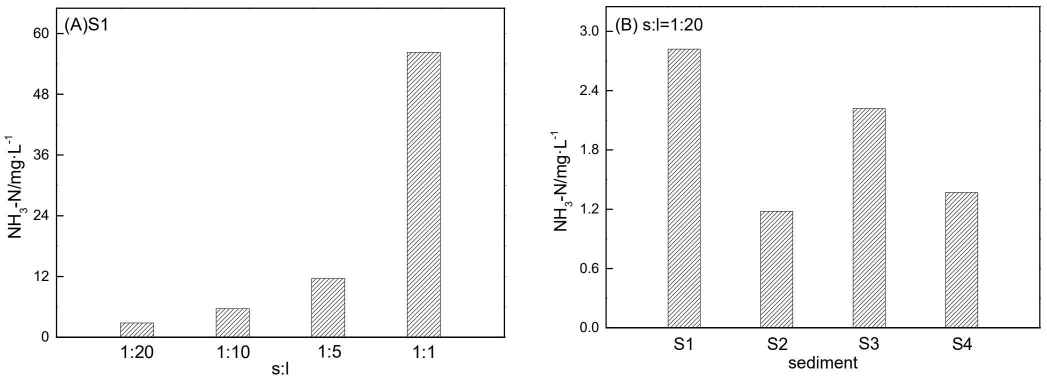

Figure S3. Ammonium concentration in ultrapure water-sediment incubation system at different sediment-to-water ratios (A) and four sediments (B) by calculating.

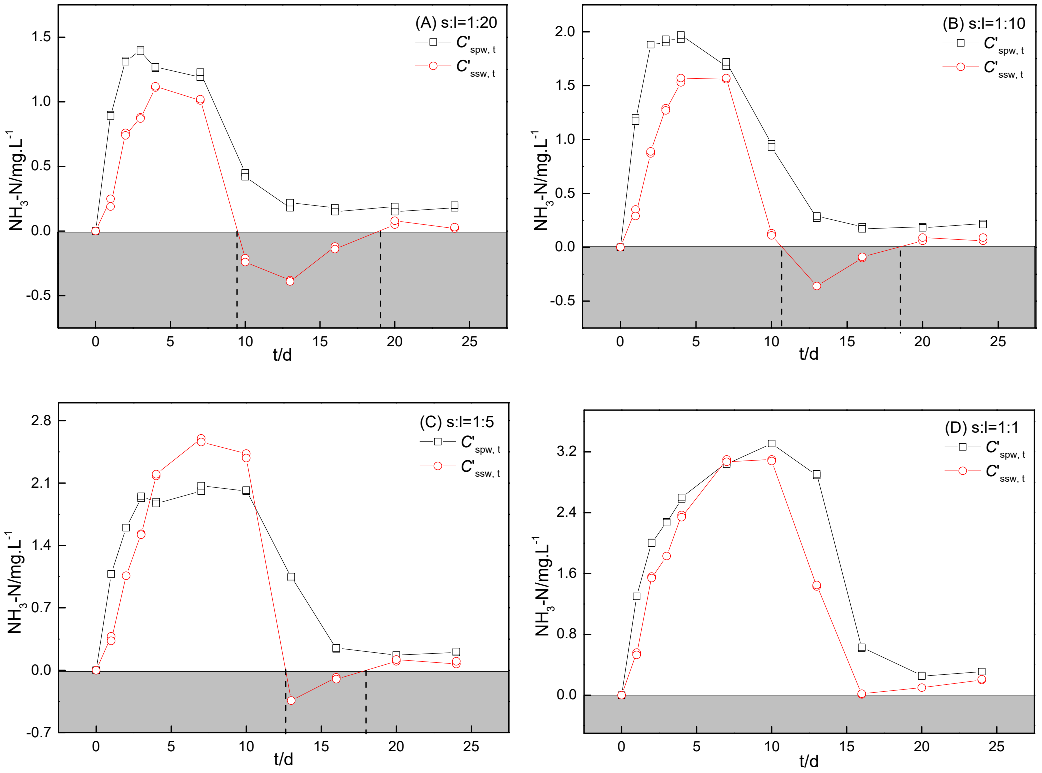

Figure S4. The intrinsic kinetics of ammonium in the sediment (S1) incubation at different sediment-to-water ratios in ultrapure water ($C'_{spw,\,t}$) and sample water ($C'_{ssw,\,t}$). The sediment-to-water ratios for (A), (B), (C) and (D) were 1:20, 1:10, 1:5 and 1:1, respectively. $C'_{spw,\,t}$ and $C'_{ssw,\,t}$ are the difference between $C_{spw,\,t}$ and $C_{pw,\,t}$, $C_{ssw,\,t}$ and $C_{sw,\,t}$, respectively.

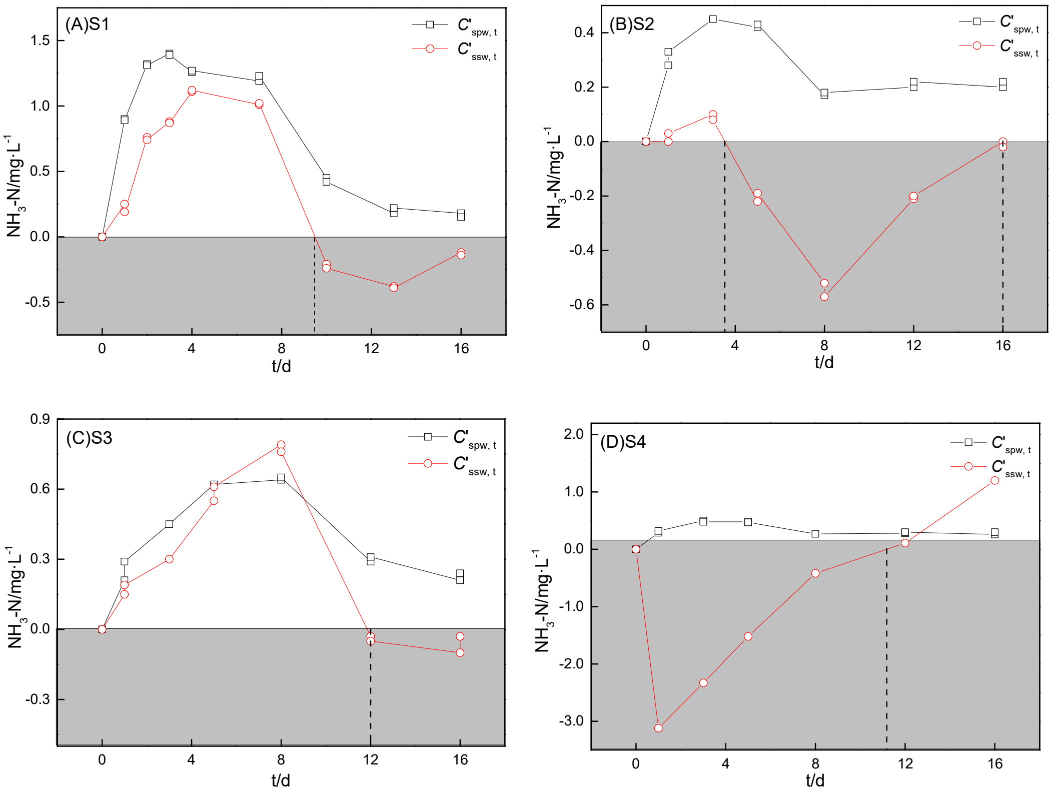

Figure S5. The intrinsic kinetics of ammonium in the four sediments incubation in ultrapure water ($C'_{spw,\,t}$) and sample water ($C'_{ssw,\,t}$). The sediment-to-water ratio was 1:20 for four sediments (S1–S4). $C'_{spw,\,t}$ and $C'_{ssw,\,t}$ are the difference between $C_{spw,\,t}$ and $C_{pw,\,t}$, $C_{ssw,\,t}$ and $C_{sw,\,t}$, respectively.
